# Moving Average-Based Multitasking In Silico Classification Modeling: Where Do We Stand and What Is Next?

**DOI:** 10.3390/ijms23094937

**Published:** 2022-04-29

**Authors:** Amit Kumar Halder, Ana S. Moura, Maria Natália D. S. Cordeiro

**Affiliations:** 1 LAQV@REQUIMTE, Faculty of Sciences, University of Porto, 4169-007 Porto, Portugal; amit.halder@fc.up.pt (A.K.H.); ana.cristina.moura@fc.up.pt (A.S.M.); 2 Dr. B. C. Roy College of Pharmacy and Allied Health Sciences, Dr. Meghnad Saha Sarani, Bidhannagar, Durgapur 713212, West Bengal, India

**Keywords:** multitasking in silico modeling, moving average approach, virtual screening, software

## Abstract

Conventional in silico modeling is often viewed as ‘one-target’ or ‘single-task’ computer-aided modeling since it mainly relies on forecasting an endpoint of interest from similar input data. Multitasking or multitarget in silico modeling, in contrast, embraces a set of computational techniques that efficiently integrate multiple types of input data for setting up unique in silico models able to predict the outcome(s) relating to various experimental and/or theoretical conditions. The latter, specifically, based upon the Box–Jenkins moving average approach, has been applied in the last decade to several research fields including drug and materials design, environmental sciences, and nanotechnology. The present review discusses the current status of multitasking computer-aided modeling efforts, meanwhile describing both the existing challenges and future opportunities of its underlying techniques. Some important applications are also discussed to exemplify the ability of multitasking modeling in deriving holistic and reliable in silico classification-based models as well as in designing new chemical entities, either through fragment-based design or virtual screening. Focus will also be given to some software recently developed to automate and accelerate such types of modeling. Overall, this review may serve as a guideline for researchers to grasp the scope of multitasking computer-aided modeling as a promising in silico tool.

## 1. Introduction

The current year marks 60 years of the onset of two-dimensional quantitative structure–activity relationship (2D-QSAR) modeling, following the pioneering work of Hansch in 1962 [1]. In fact, Hansch’s work has paved the way for computer-aided drug design endeavors that, since then, have been enriched by several other ligand-based (e.g., 3D-6D QSAR, pharmacophore mapping, etc.) and structure-based (e.g., molecular docking, molecular simulations, homology modeling, etc.) methodologies [2,3,4]. However, the advent of these relatively new in silico approaches does not definitely extinguish the relevance of 2D-QSAR modeling in computational chemistry [5]. Rather, owing to its simple and versatile nature, the practice of 2D-QSAR modeling has been expanding and is applied now to numerous different areas of science such as nanotechnology, materials, environment, and so forth [2]. Even for the drug discovery and development process, where the researchers have many other in silico alternatives, 2D-QSAR still offers fast and effective solutions [6,7]. While the primary objective remained unchanged, i.e., the consistent prediction of response variable(s), it is undeniable that the past few decades have witnessed a variety of progress in concepts and applications of 2D-QSAR modeling [3]. Modern 2D-QSAR practices now embody a set of in silico modeling tools in which statistical and/or machine learning techniques are applied to derive relationships between the targeted response variable(s) and the descriptors encoding molecular structural attributes and properties. Naturally, the reliability of these in silico modeling tools largely depend on the size and diversity of the datasets employed [2]. Indeed, due to the steady growth of available data, ‘big-data’ became a new trend for in silico modeling tools that in turn have been fueled by numerous advances in computational efficiency as well as in model development strategies. However, resorting to big data does not always ensure an improvement of the applicability of the derived models since variations in the experimental (or theoretical) conditions used to determine the response variable(s) are often ignored [8]. That is why strategic data integration-based in silico modeling approaches appear promising [8,9,10], mainly because they do not only help to increase the size and diversity of the targeted data but at the same time, account for the variations that are frequently encountered while merging data collected from several sources [11,12]. Multitarget or multitasking (mtk) in silico modeling is a comparatively novel advanced strategy that allows the merging of datasets pertaining to multiple conditions to simultaneously predict the response variable(s) under such diverse conditions [2,13,14,15].

The present review covers the main aspects of multitasking classification modeling based on the Box–Jenkins moving average approach, since the latter has emerged as one of the simplest approaches for building up unique 2D-QSAR models from large heterogeneous datasets with multiple features [15,16]. Apart from discussing the objectives, methodologies and applications, the review also sheds light on the software recently developed for supporting and facilitating mtk-QSAR analyses. The future scope of such multitasking modeling is also discussed in detail. It is important to mention here that the application of perturbation theory along with machine learning techniques (PTML), as well as those combined with the information fusion technique (PTMLIF), also falls within the scope of the Box–Jenkins-based multitasking modeling, but such methodologies have been thoroughly reviewed very recently [10] and have thus been excluded to maintain brevity of this review.

## 2. Multitasking QSAR Modeling: Rationale and Existing Challenges

In silico QSAR modeling stands for the common practice of looking for relationships between the endpoint (EP) response(s) of interest and descriptors encoding the molecular structures (S) and properties (P) of a set of chemicals, through multivariate statistical methods and/or machine learning techniques [2,17]. These relationships aiming at either classification-based or quantitative predictions of the response(s) values pertain then to mathematical models as follows:

EP = f (S,P) (1)

in which EP is considered to be only a function of S and P. However, it is well known that the response(s) values clearly depend on the type of experimental procedures or theoretical calculations employed or even if following the same type of protocol but in different conditions (C). Therefore, with a larger perspective, the mathematical models are to be described as follows:

EP = f (S,P,C)(2)

The workflow usually tracked for setting up QSAR models begins by assembling the chemicals with known EP response(s) to form a dataset and in so doing, one frequently encounters very dissimilar conditions. Conventional QSAR modeling, often referred to as ‘one target’ or ‘single-task’ in silico modeling, relies primarily on assuming similarity in the experimental and/or theoretical conditions that often ends up limiting the inclusion of small datasets. Even if datasets with multiple experimental/theoretical conditions are being included, the influence of their variation is frequently ignored. As explained hereafter, the moving average-based multitasking (MA-mtk) in silico modeling tends to overcome such limitations [12,13].

Firstly, small datasets with large variations in the experimental and/or theoretical protocols followed can be conveniently accommodated in the modeling datasets, thereby enhancing their diversity as well as the applicability of the following in silico models. Therefore, a shortage of data for a specific experimental/theoretical condition does not pose any challenge for the modeler. This may be exemplified from a recently reported study [18], in which the primary objective was to characterize the cytotoxicity of acrylic acid-based dental monomers through QSAR modeling. The maximum number of data points found for compounds assayed against one single type of cell line was 39, using the same biological measurement, but when the cytotoxicity against other cell lines ought to be included, a dataset of 138 data points could be built. Since this larger dataset included information pertaining to as many as 18 different cell lines probed by five types of measurements, it seemed worthwhile resorting to a moving average-based multitasking approach to retrieve a more reliable QSAR model as compared to a single-task model, aside from covering a larger response and experimental space [19].

Secondly, one unique mtk-QSAR model is capable of predicting multiple outcomes simultaneously and the models’ descriptors may serve as ‘global descriptors’ to derive general and holistic mechanistic interpretations for the endpoint response(s), which may be disconnected on the grounds of conditions but are likely to be linked with each other regarding mechanism(s) of action [8,20]. Finally, multitasking QSAR modeling improves the scope of virtual screening since the virtual hits that are obtained display predictivity against several different conditions. Therefore, the modelers are left with the options to select those hits that are predicted to have positive responses either against all/maximum conditions (for the design of pan-inhibitors, for example) or against some specific conditions (e.g., for choosing isoform specific inhibitors) depending on the aim of the investigation [21,22].

Nevertheless, one may face several challenges while developing moving average-based multitasking in silico models as not only additional steps are involved but additional statistical and validation criteria are also to be satisfied, when compared to conventional ‘single-task’ in silico modeling [19]. It must be emphasized that similar to conventional QSAR approaches, MA-mtk modeling follows the best practices outlined by the Organization for Economic Co-Operation and Development (OECD), which state that each model should have a defined end point, an unambiguous algorithm with a defined domain of applicability, goodness of fit, robustness and predictive ability, as well as a mechanistic interpretation, if possible [23]. The main challenge lies in the fact that the decision regarding some choices may vary from one investigation to another and a general consensus is difficult to achieve. For example, in the initial stages, the modeler needs to decide how many experimental and/or theoretical conditions are to be taken into account for the modeling. Variations in the biological targets, types of biological measurements, and experimental protocols are often easily identified as possible experimental conditions but such selection may vary depending on the investigation purpose. Furthermore, such selection of experimental (theoretical) conditions often depends on the availability of information in the literature or databases.

On the other hand, since the moving average-based mtk-QSAR approach discussed in this review aims at developing classification-based models, the selection of acceptable cut-off values for the response variable(s) becomes crucial. Naturally, such selection is expected to vary in different studies and research. Recently, Kleandrova et al. recommended that for drug-design purposes, the selected cut-off values should be set at least at the sub-micromolar level and at the same time, the chosen cut-offs should prevent any excessive imbalance between the number of chemicals assigned as active/positive and those assigned as inactive/negative [24]. Another key challenge is to ensure that the predictivity of the so derived models may be overestimated on the basis of some experimental and/or theoretical conditions. The chance is higher because this type of in silico modeling constitutes a single computational framework that yields a unique model and thus, it is likely that low predictive accuracy against some conditions is overshadowed by the high accuracy over other conditions. Ideally, the model should uniformly predict all experimental and/or theoretical conditions. To tackle this challenge, the ‘condition-wise prediction’ strategy has recently been introduced since it readily provides predictions of the model versus each considered condition [12,25]. After carefully checking the results, the conditions with poor prediction statistics may be identified as outliers and at least a warning may be provided to avoid making predictions with such conditions [18,21,25].

## 3. Multitasking In Silico Modeling Methodologies

### 3.1. Moving Average Approach

The moving average (MA) approach is fundamental for developing multitasking models through data-integration, including PTML-based modeling efforts [10,13]. In such an approach, the descriptors are transformed in a way so that these encode information about both the compounds’ structures and the experimental/theoretical conditions under which their response variable(s) have been attained. Therefore, even if the same chemical compound displays two different endpoint responses pertaining to two different conditions, the MA approach must generate two different descriptors for it. Originally, the Box–Jenkin’s moving average (BJMA) approach has been delineated for time-series analysis, that is, based on computing successive average values of a defined system property to forecast its value at a different time [13,26]. In MA-mtk modeling, the Box–Jenkin’s operation is not related to the time domain but instead rather to the various targeted experimental and/or theoretical conditions. Even though a range of BJMA schemes have so far been employed, all of these originated from the following formula:

∆(*D_i_*)*c_j_* = *D_i_* − *avg*(*D_i_*)*c_j_* (3)

Therefore, the new descriptors ∆(*D_i_*)*c_j_*, also referred to as ‘deviation descriptors’, are calculated by subtracting from the input descriptors (*D_i_*) the avg(*D_i_*)*c_j_* values, in which the latter stands for the arithmetic mean of active/positive data points of a specific element of the experimental and/or theoretical conditions (ontology). Along with ∆(*D_i_*)*c_j_*, the *avg*(*D_i_*)*c_j_* values have also been used as descriptors in previous works [27,28]. Other operators that have recently been employed are basically modified forms of this formula, corresponding to normalizations of the original operator with respect to the variation in sample size against each condition and/or *D_i_* values (see Table 1). Still, there is not enough evidence to prove that these modified operators may actually give rise or not to better statistical models than the original operator. In a recent study, a comparative analysis was carried out using some of these modified BJMA operators but a large variation in the predictive accuracy of the derived models was not observed [12]. Yet, the importance of such modifications in developing more predictive models may not be ruled out entirely.

### 3.2. Descriptor Calculation

The role of the original descriptors (*D_i_*) should not be overlooked in multitasking QSAR modeling even though these are transformed by BJMA. Note that due to the nature of the moving average approach already discussed, descriptors with high variances should be preferred for modeling since near-constant descriptors may fail to provide information about the experimental systems. This is why a number of mtk-QSAR models were developed using 2D-atom and bond-based topological, as well as 2.5D chiral algebraic molecular descriptors. Open-access Java-based tools such as QuBiLS-MAS and MODESLAB have been extensively employed to develop mtk-QSAR models since these software tools allow the computing of a large number of unique graph-based topological descriptors [36,37,38]. Software such as Dragon [39] has also a long history in setting up QSAR models with a large number of descriptors that belong to various categories (e.g., constitutional, atom-based fragments, geometrical, topological, etc.). Moving average-based multitasking QSAR modeling is no exception and several models have been reported lately, using such DRAGON descriptors. As such, it can be judged that there are no restrictions on the type of descriptors to be employed in MA-mtk in silico modeling but no matter what type of descriptor is used, pre-treatment is required to remove the near-constant descriptors.

Here, it is important to mention that even though moving average approaches help in merging the information pertaining to experimental conditions with that of the original descriptors *D_i_* for jointly handling structural and physicochemical properties, the mechanistic interpretation of the new descriptors ∆(*D_i_*)*c_j_* becomes much more complicated. Simply put, these new descriptors explain the contribution of the original descriptors *D_i_* with respect to the experimental elements *c_j_*. In fact, ∆(*D_i_*)*c_j_* descriptors built with the same *D_i_* values but with different experimental elements (*c_j_*) were found in some models. From one side, these models are clearly justified by the ability of such variables towards usefully predicting the desired endpoints, though with costs regarding their mechanistic interpretation. For example, in one work [21] two different descriptors ∆(C-012)*_me_* and ∆(C-012)*_bt_* appeared in the same model with opposite correlation with the response variable. Therefore, it was inferred that the molecular fragment descriptor C-012 improves the biological activity when it is associated with the experimental condition—a kind of measurement of effects (*m_e_*), whereas it deteriorates it when the same descriptor is related to another experimental condition as the assay types (*a_t_*). Similar to conventional QSAR modeling, models containing a smaller number of simpler descriptors are preferred. For example, in a very recently published study [40], the authors developed two non-linear models with almost similar statistical results. One of these was preferred over the other since it consisted of a smaller number of descriptors and, at the same time, it provided a simpler and more detailed mechanistic interpretation for the dataset.

### 3.3. Data Pooling, Databases, and Inclusion/Exclusion Criteria

Dataset collection and curation are undeniably crucial in MA-mtk in silico modeling. Whenever endpoint responses are aimed to be modeled, one may rely upon databases of compounds, such as the databases ChEMBL (https://www.ebi.ac.uk/chembl/), BindingDatabase (https://www.bindingdb.org/bind/index.jsp), or AFLOW (http://aflowlib.org/), for a quick retrieval of data points. However, often datasets are required to be manually collected from the literature. Unlike conventional in silico modeling, MA-mtk modeling incurs risks when merging data points coming from diverse experimental and/or theoretical conditions and therefore, the curation of the datasets needs to be carefully performed. That is, one specific compound may be placed in the dataset for MA-mtk modeling multiple times only if it leads to data points pertaining to different conditions. If one compound is found to have the same categorical end-point with respect to the same experimental and/or theoretical conditions, only one data point is retained, for obvious reasons. However, given the same experimental/theoretical conditions, if two different categorical end-points (for example, one active and another inactive) are found, both such data points should be excluded to avoid inconsistent outcomes. In the latter case, it is always better to fully inspect the reported investigations where such large variations in results have been obtained and try to address what could have caused the variations before including their data in the modeling dataset.

### 3.4. Dataset Division

Another important consideration is, of course, in regard to dataset division. In one approach, the entire modeling set is used for deriving the models and then the dataset is divided into a training and a prediction set [41,42,43]. Alternatively, a second approach may be adopted where the dataset is first divided into a modeling set and an external validation set. The modeling set is only used for computing the *avg*(*D_i_*)*c_j_* descriptors and subsequently the values of those are used in the calculation of ∆(*D_i_*)*c_j_* descriptors for both the modeling and the external validation set [11,12,18]. In this second approach, the external validation set has no role either in the model development or descriptor generation, and thus it can be regarded as a true validation set. Noticeably, in such an approach, *avg*(*D_i_*)*c_j_* is fixed and any new compound can then be directly fitted with the developed model for its prediction. One should note, however, that the modeling dataset may be further divided into a training and a test set, where the latter may serve multiple purposes. Firstly, it can act as an additional validation set and if similarity is reached regarding the predictive accuracy between this test and the external validation set, that thereby further justifies the consistent prediction of the models, irrespective of which dataset division approach is adopted. Actually in a previous work, we found that, for most cases, the predictive accuracy of test and external validation sets are remarkably close to each other. On the other hand, such a test set may also serve as a calibration set for selecting the best model out of many possible. Some methods, such as the PS3M later described or hyperparameter optimization for machine learning techniques, often require calibration and the test set may thus be utilized to ensure better performance of these techniques [11,12,18,25].

### 3.5. Set-Up of the MA-Mtk Model

Undoubtedly, robust model development strategies are required for setting up moving average-based mtk-QSAR models, since the number of input descriptors are actually multiplied based on the number of experimental conditions. Due to the same reason, effective variable selection procedures should be employed for building linear or non-linear interpretable models with a limited number of features. Forward selection strategies such as the fast stepwise selection algorithm have been successfully employed initially to develop linear discriminant analysis (LDA) models using commercial software packages [27,37]. However, a more advanced stochastic approach, such as the genetic algorithm, later proved to be an extremely useful alternative and was applied on the open source software QSAR-Co for setting up LDA-based mtk-QSAR models [44]. Recently, two non-stochastic approaches, namely the fast stepwise (FS) and sequential forward selection (SFS) algorithms, were available for establishing LDA models in another open source software QSAR-Co-X [12]. Both stochastic and non-stochastic strategies have their advantages and disadvantages. For example, the stochastic GA variable selection approach lacks reproducibility and it is not known a priori how many runs are needed to reach the best LDA model, meaning that it might be needed to be repeated several times [12]. Nevertheless, due to its unique feature selection methodology, the chance of obtaining a highly predictive LDA model with GA is remarkably high, especially when other strategies fail to develop predictive models from a large number of independent parameters. In contrast, with the same parameter settings, the FS or SFS variable selection algorithms are always reproducible and the corresponding LDA models are also easily obtained. Yet, no feature selection algorithm is flawless and comparative analysis may be the only way to retrieve the most predictive linear model [30]. Very recently, a post-selection similarity search-based modification strategy (so-called PS3M) has been proposed with the hypothesis that, no matter what variable selection algorithm is employed, the model produced should be treated as a reference model that itself is not the best model but it should be similar to the best model [18]. As such, descriptors which are similar or highly correlated to each descriptor of the model are firstly searched using a Euclidian distance scheme. Subsequently, each original model descriptor is replaced with its similar descriptors found and the resulting modified models checked to see if they have better statistical quality or not. If a better model is obtained, it is automatically treated as a reference model and the same steps repeated until no better model is obtained. As of now, PS3M appears as a promising strategy, especially when a large pool of descriptors is employed and therefore its potential in mtk-QSAR modeling may not be ignored [18,21]. Apart from the selection schemes referred, the Shannon entropy has also been used in research for the most discriminating features to set-up non-linear models [33,45]. Even though non-linear models developed with a maximum pool of descriptors, the latter abolishes the mechanistic interpretability and, therefore, feature-selection strategies are often employed to establish models with a limited number of variables that afford highlighting of the most significant descriptors. Similarly, several advanced machine learning (ML) tools have been applied to search the most predictive non-linear models (see Table 2), which at the cost of mechanistic interpretability produce highly predictive mtk-QSAR models [12,25]. So far, ML techniques such as artificial neural networks and tree-based techniques such as random forests (RF) and gradient boosting have proven to be the most successful ones [18,21,24,33,41,42,43,46]. In a recent work, even though deep neural networks gave rise to a highly predictive model its predictivity was similar to the RF model, which was ultimately reported [21]. Thus, deep learning may play an important role in future developments of MA-mtk models [2]. Due to the complex nature of the data-matrices involved in this type of in silico modeling, it is always advisable that along with deploying multiple ML strategies, hyperparameter tuning should also be taken into consideration for optimizing the parameters to obtain the validated models [12]. For example, in one recent investigation [18], six different ML methods (i.e., RF, GB, SVM, *k*NN, NB and ANN) were employed with hyperparameter optimization to develop non-linear models and it was the ANN model that afforded the most predictive model. In another study [25], seven different ML methods were employed with hyperparameter optimization, and the internal predictivity (confirmed by 10-fold cross-validated accuracy) was as follows: GB (91.54%) > RF (91.02%) > DT (84.33%) > ANN (82.97%) > *k*NN (79.20%) > NB (69.40%) > SVM (62.30%). It is noticeable that even with hyperparameter optimization, large variations in predictivity may be observed when one switches from one ML tool to another. Therefore, the application of multiple robust ML methods definitely improves the scope of reaching better predictive models.

### 3.6. Statistical Analysis and Validation

The statistical quality of both linear and non-linear mtk-QSAR classification models can be judged in terms of the criteria goodness-of-fit and goodness-of-prediction. Goodness-of-fit is frequently checked by standard statistics such as the Wilks’ lambda (λ), chi-square (χ2), the Fisher ratio (*F*), and the corresponding *p*-level (*p*). Similarly, the predictive accuracy of the models is commonly estimated by means of the confusion matrix that comprises the number of true positives (TP), true negatives (TN), false positives (FP), false negatives (FN), and allows then to compute other statistics such as the accuracy (Acc), the Matthews correlation coefficient (MCC), and so forth [25,44]. The moving average methodology generally gives rise to highly correlated modified variables and data pretreatment is thus required to remove such redundant features. In particular, the proposed linear model should also be assessed for chance correlation by the *Y*-randomization test [44], recently modified for mtk-QSAR modeling to also consider the role of experimental conditions (*c_j_*). In this modified test, so called *Y* randomization with conditions (*Y_c_*) [12], the response variable(s) along with the experimental elements are scrambled to generate multiple randomized data-matrices. New models, based on the fits to these scrambled data-matrices, are then calculated using the same original model descriptors. A high difference between the statistical parameters (i.e., λ and Acc) of the new models and the original model then conveys its robustness [12]. The range of validity of a QSAR model must be well assayed, in terms of the range of biological response data within, it will predict reliably and also in terms of the type of chemical structure on which it is based.

No in silico QSAR model is meant to predict the whole range of possible chemicals and targeted response(s). That is, any QSAR model must have a defined applicability domain (AD). From the viewpoint of mt-QSAR modeling, the AD is the endpoint response(s) and experimental (theoretical) space within which the model can make trustworthiness predictions. A number of strategies have so far been applied to define the AD of QSAR models and none of these has proven to be superior to others [47]. Two different ways may be used to determine the applicability domain of the MA-mtk models. The first one is essentially defined by the experimental elements since it is always advisable to consider only external validation compounds that follow the same experimental and/or theoretical conditions under which the modelling dataset samples have been obtained. Structural outliers are generally identified through the same procedures by which conventional classification-based QSAR models are (e.g., the leverage approach). However, AD determination methods may vary depending on the type of model, i.e., linear or non-linear models. For linear models, the AD set by the standardization method proposed by Roy et al. [48] has lately been applied in several studies [11,12,18]. In contrast, the AD of non-linear models is difficult to define but techniques such as the confidence estimation approach [49] can be used to identify structural outliers [12,44]. Recently, another method for establishing the AD of any type of model has been suggested, in which local binary scores are calculated for its descriptors based on their minimum and maximum values. Subsequently, these scores are summed up to obtain a total score from which the outliers are detected as the latter should have a total score less than this [38].

## 4. Applications of Mtk-QSAR Modeling

A considerable number of mtk-QSAR models based on the moving average approach have been developed and proposed in the last 10 years for tackling a wide range of applications, such as drug design and development, toxicology, and environmental sciences, including nanotechnology. For the sake of discussion, in this section, such mtk-QSAR models will be divided into two main categories considering the main objective of the study, namely as targeting the activity against cells, organisms, and species (a), or that against bio-macromolecular targets (b). There are however some investigations that fall into both categories [28,43,50,51,52,53]. Most of these models have been developed by collecting data from the CHEMBL database, which is regarded as one of the largest and most reliable databases to date.

### 4.1. MA-Mtk Modeling of the Activity against Cells/Organisms/Species

Due to a complex bio-functional mechanism, the activity of a compound may vary from one cell to another as often observed in research focused on the anticancer or antimicrobial properties of chemicals [27,33]. Therefore, a major focus has been invested in applying multitasking modeling for predicting the antiproliferative or antimicrobial activity of compounds against various cells (i.e., mammalian or microbial).

**Figure 1:**
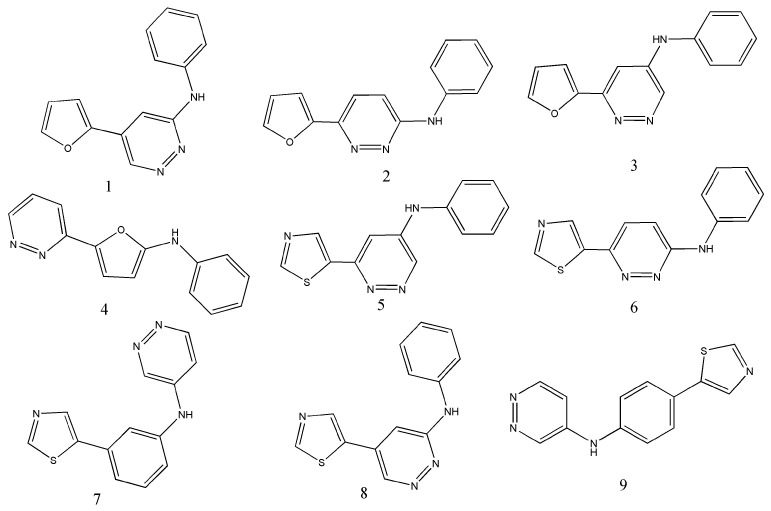
Examples of new anti-breast cancer leads suggested in the mtk-QSAR modeling study by Speck-Planche et al. [55].

Let us start with a fragment-based mtk-QSAR modeling study reported in 2011, based on a dataset containing 449 compounds with measured cytotoxicity against twelve different mammalian sarcoma cells (making a total of 3017 data points) [27]. In this study, just one particular category of descriptors was employed, namely substructural descriptors comprising functional group counts, atom centered fragments and spectral moments of the bond adjacency matrix. Among these, only the spectral moment descriptors were subjected to the moving average approach to derive *avg*(*D_i_*)*c_j_* as well as ∆(*D_i_*)*c_j_* descriptors. Combining these different classes of descriptors and by adopting a linear discriminant analysis (LDA), an interpretable model with a pool of thirteen descriptors was finally built that demonstrated consistent accuracy of ca. 91 and 90% over the training (*N*_training_ = 1887) and test (*N*_test_ =1130) data-point sets, respectively. A similar methodology was followed in the next few years to develop predictive mtk-QSAR models based on datasets of chemicals with tested antiproliferative potential against prostate carcinoma cells [54], breast carcinoma cells [55], gliomas [56], colorectal carcinoma cells [57] and bladder cancer cells [58]. A couple of remarks from these studies are picked out here. First, these mtk-QSAR models have always been derived by resorting to deviation descriptors. Second, in some of the later studies, an artificial neural network (ANN) methodology has also been employed to set-up non-linear models with selected features. In fact, the non-linear models obtained through including a larger number of ∆(*D_i_*)*c_j_* features displayed a higher predictive accuracy as compared to their LDA counterparts. These remarks clearly indicate the significance of ∆(*D_i_*)*c_j_* descriptors. Furthermore, despite the fact that such models were based on a considerably large number of data points, all depicted an overall predictive accuracy higher than 85% and the majority of them attained an accuracy above 90%. Worth mentioning as well here, is that the antiproliferative potential of chemicals is a difficult biological property to target from a computer-aided modeling point of view, due to the fact that numerous biochemical mechanisms may be involved. Therefore, from this aspect, the performance of all these models should be considered as highly satisfactory. What is more, one should highlight the advantage of resorting to fragment-based descriptors in mtk-QSAR modeling, that is, the possibility of estimating the contributions of different fragments to the biological activity studied that can be employed as 2D pharmacophores for designing new possible leads. As an example, Figure 1 shows new anti-breast cancer leads suggested following that strategy, based on their fragment contributions [55].

More recently, Kleandrova et al. reported a multitasking modeling study with the aim of simultaneously predicting the inhibitory activity of chemicals against various liver cancer cell lines [32]. The dataset used in this study was collected from the Genomics of Drug Sensitivity in Cancer (GDSC) and contained 192 (FDA approved or experimental) drugs that have been assayed against 17 different liver cancer cell lines, resulting in a total of 3079 data points. Furthermore, only ∆(*D_i_*)*c_j_* descriptors derived from total and local (atom-based) non-stochastic quadratic indices were chosen to build non-linear classification models using ANN. The best mtk-QSAR model found contained nine descriptors and gave rise to a moderate predictivity (ca. 85% overall accuracy) but enabled the virtual design of six new promising anticancer agents against the liver cancer cell lines considered.

A significant number of multitasking modeling studies based on the moving average approach have been performed to probe antimicrobial and antiviral activities in the last 10 years [31,50,51,52,53,59,60,61,62,63,64,65,66]. Table 3 displays the details of the methodology employed, the studied endpoint responses, and bio-targets considered, per year. While the evidence of a preference for ANN-based models is observed in certain instances, FS-LDA is the most usual choice for the methodological approach to be followed. The type of endpoints investigated also covered a wide range apart from the antimicrobial or antiviral activities, ranging from solely toxicity properties to absorption, distribution, metabolism, elimination, and toxicity (ADMET) since the latter play a key role on guiding hit-to-lead and lead-optimization efforts, and on average the predictive accuracy for these models was in the proximity of 90% or greater.

However, some of the works had particular aspects that needed more detailed address. Regarding antimicrobial peptides (AMP) [50,64], since they have a unique structural nature, as an approach to effectively compute the challenging AMP molecular descriptors, the peptide sequences were first converted to FASTA sequences, which were subsequently converted to 3D formats for the calculation of topological indices (e.g., Kier–Hall indices and Broto–Moreau autocorrelations) that were then subjected to the BJMA technique. Both works adopted the strategy of collecting AMPs from the Database of Antimicrobial Activity and Structure of Peptides (DBAASP), and the models exhibited a predictive accuracy higher than 90% in both the training and prediction sets.

Another team established as its main goal to generate complex networks of AIDS incidence among USA counties, relative to the preclinical activity of drugs against the human immunodeficiency virus (HIV) [62]. Several ANNs have been trained for such a purpose, using as input information the indices of social networks (taken from public epidemiological databases) and molecular graphs (i.e., Balaban information indices to describe the chemical structures of anti-HIV drugs). The best mtk-QSAR found was a linear ANN and exhibited an overall accuracy of ca. 80%. Moreover, the drug–county network built from such a model supplied useful information about the most effective drugs to treat HIV in different populations (from the US counties) with a given epidemiological prevalence.

Another work that was published more recently, in 2017, is also worth mentioning here [31]. Given the fact that Hepatitis C is one of the deadliest, unresolved health problems globally, the modeling addressed both the anti-Hepatitis C potency and ADMET profiles of several chemicals collected from ChEMBL by considering also their testing conditions, namely: the types of biological measurements, different bio-targets, information regarding the assays (labeling whether the assay focused on the study of binding phenomena, functional/physiological responses, or ADMET profiles), as well as the involved target mappings. Furthermore, this work, apart from considering the latter experimental conditions, modified the moving average formulae by multiplying the deviation descriptors with a probabilistic factor (*p_c_*) denoting the degree of reliability of the experimental assay (i.e., autocuration, intermediate, and expert, respectively). As such, the best FS-LDA-based linear model found (40,158 data points), developed with topological descriptors known as bond-based quadratic indices [36], afforded an overall accuracy higher than 95%.

Notwithstanding, all of the above-mentioned investigations developed the MA-mtk models with relatively large datasets, something that was not the case for two recent reports, one that involved the environmental toxicity of deep eutectic solvents [66] and another about the cytotoxicity of acrylic acid-based dental monomers, this latter with only 138 data points [18]. As the last dataset was highly inhomogeneous in nature, given the fact that 58 different chemicals were tested against 18 different cell lines with five different types of measurements, apart from model generation, the challenges which also remained were the validation of the model and to establish that each experimental condition of the dataset was predicted with consistent accuracy. The development strategy involved the selection of the best mtk-QSAR model from twelve different linear models generated by varying data-distribution and feature-selection techniques. The most predictive linear model was generated with moderate to good predictivity against the training (91%), test (91%) and external validation (85%) sets. Note that, in this work, the PS3M strategy was employed for the first time, and it led to a ‘similar’ model with higher statistical accuracy against the training (94%) and the external validation (89%) sets without any change in the predictive accuracy of the test set. Finally, a technique named ‘condition-wise prediction’ was employed to split the prediction results into different experimental conditions in order to identify poorly predicted experimental conditions. However, it was observed that the model provided satisfactory predictivity against most of these conditions. Such investigations show that the MA-mtk modeling approach may also be applied to relatively small datasets but many more of these studies should be reported in the future along with experimental validation to confirm this idea.

To conclude this analysis of MA-mtk modeling of the activity against cells/organisms/species, going beyond the scope of drug design and discovery, some MA-mtk studies have also focused on the environmental toxicity of diverse categories of chemicals [28,33,67]. Particularly noteworthy is a recent multitasking modeling study of the ecotoxicity of various classes of pesticides [33]. Departing from 260 structurally diverse peptides, a dataset containing 3610 data points was formed by considering four primary different experimental conditions, namely: *m*_e_ (toxicity measurements), *b_s_* (bioindicator species), *a*_g_ (assay guidelines) and *e*_p_ (exposure periods). Alongside these, three secondary additional experimental conditions, i.e., concentration lethality (*l*_c_), target mapping (*t*_m_) and time classification (*t*_c_), were also considered for computing the moving average-based descriptors. The ANN model with the highest discriminant power found thereafter included nine deviation descriptors, computed from the original graph-based topological features, and depicted an overall accuracy of 83 and 76% in the training and prediction sets, respectively. The same dataset and starting descriptors were later used in another investigation [12] for building multitasking models using a different machine learning tool—i.e., random forests (RF), but in particular with the aim of comparing different moving average-based algorithms to understand their effects in the models’ predictive accuracy. Five different moving average algorithms, which had been employed previously in different investigations, were used to derive five different models. Interestingly, the comparative analyses showed that the predictive accuracy of these models did not vary to a large extent.

### 4.2. MA-Mtk Modeling of the Activity against Bio-Macromolecular Targets

As different pathways and bio-macromolecular targets have increasingly been identified in the last few decades, MA-mtk modeling is becoming an interesting tool in the design of both selective and pan-inhibitors, depending on the roles of these closely related bio-macromolecular targets against any specific disease. Quite expectedly, significant efforts have since been invested to set up mtk-QSAR models with multiple macromolecular cellular targets [38,68,69,70]. For example, in 2013, with a strategy encompassing LDA and ANN tools to set up linear and non-linear models for probing several proteins involved in the progression of leukemia, the substructural and global descriptors that were used had no modification but spectral moments derived from the bond adjacency matrix (µ*_k_*) were subjected to the moving average approach to compute the deviation descriptors [69]. Both ANN and LDA models had an overall accuracy above 90%, with the ANN model comprising a total of eleven descriptors out of which four belonged to ∆(*D_i_*)*c_j_*. Another study by Casañola-Martin et al. [70] is worth mentioning here. Starting from 2954 unique drugs retrieved from the CHeMBL database making 5062 data points, the authors developed an LDA-based mtk-QSAR model with seven descriptors that predicted the outcomes of more than 450 different type of assays against at least 1 out of 20 experimental parameters related to the ubiquitin–proteasome pathway. Though affording an overall accuracy of 70%, when considering the complexity of the modeling-data matrix as well as the fact that numerous biochemical mechanisms are likely to be involved in the ubiquitin–proteasome pathway, this study clearly demonstrated that the moving average-based mtk-QSAR modeling may be expanded towards overly complex biochemical pathways.

**Figure 2:**
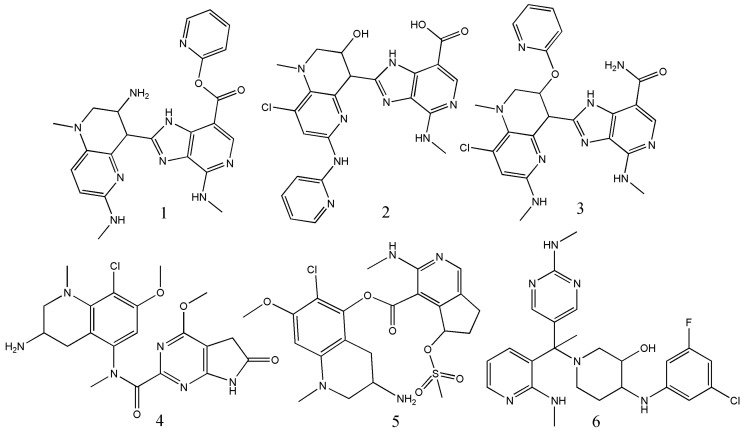
Promising BET bromodomain inhibitory leads proposed in the mtk-QSAR modeling study by Scotti and co-worker [38].

More recently, several works have focused on targeting the inhibition of various bio-macromolecular targets of cancer such as PI3K [11], AKT [25], ERK [46], MNK [21], HSP90 [41], and BET bromodomain [38]. Their main aim was to set-up models capable of simultaneously predicting the inhibitory potential of the chemicals against various isoforms of such biological targets. Notwithstanding, these models may definitely be used for obtaining isoform-specific inhibitors. Integrating other in silico strategies, especially structure-based techniques, when bio-macromolecular targets are involved can provide that aspect. In fact, recently linear and non-linear MA-mtk models were developed for coping with the inhibitors of three different isoforms of the BET (bromodomain and extra-terminal) family of bromodomain-containing proteins (i.e., BRD2, BRD3, BRD4) that serve as epigenetic regulators in the progression of cancer [38]. High accuracy values (>85%) were obtained for both models though the ANN-based model was definitely more predictive. In addition, not only the desirable fragments were identified for the design of potential virtual leads against these targets, but also the designed leads were separately docked into the active sites of X-ray crystal structures of BRD2, BRD3, BRD4 to find the most promising candidate among these leads (Figure 2).

**Figure 3:**
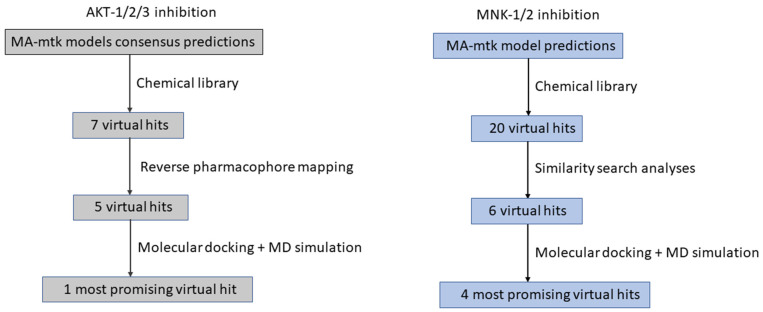
The virtual screening strategy adopted for the design of pan-AKT inhibition (**left**) and pan-MNK inhibition (**right**) [25].

The approach of mixing or coupling in silico strategies is a key aspect in these MA-mtk-QSARs. Support from other ligand- and structure-based in silico methodologies may assist in further filtering and ranking the positive hits obtained from the QSAR model (Figure 3), as the latter action cannot be provided by simple virtual screening. Furthermore, assessment of the druggability and synthetic accessibility may also help in curtaining the number of hits [8,21,24]. Table 4 lists some of these tools and webservers that have already been used along with MA-mtk models to select the hits.

Though the choice of an in silico strategy to be adopted depends largely on the researchers, more often it is the nature of the biological targets involved in the work that becomes the most crucial factor for choosing which method is to be adopted. Discussing two recently published investigations, one involving the design of pan-AKT inhibitors [25] and the other one focused on the design of pan-MNK inhibitors [21], may enlighten how rigorous application of in silico methods helps in the selection of the promising hits (see Figure 3). Being kinase enzymes, both AKT and MNK possess highly flexible catalytic sites and therefore semi-rigid docking may be unreliable. Therefore, in both these investigations, MD simulations of ligand-receptor complexes was chosen as the last resort to finalize the hits. For AKT inhibitors, virtual hits obtained from the predictive linear (GA-LDA model yielding an overall accuracy > 88%) and non-linear (developed with XGBoost yielding an overall accuracy > 91%) MA-mtk models were further filtered through reverse pharmacophore mapping strategy, i.e., the pharmacophores generated on each query compound were matched with a large database containing structure-based pharmacophores generated with the X-ray crystal structures of ligand-receptor complexes to rank these complexes as per the fit values. As such, a reverse pharmacophore mapping strategy may be exploited to validate the results of virtual screening and for filtering the hits. Here, seven virtual hits were obtained from MA-mtk modeling but five hits were retained after pharmacophore mapping for further processing. Finally, MD simulations were carried out with each of these five hits to ensure the theoretical binding potentials of these hits against all AKT isoforms and on the basis of these analyses, one candidate was selected as the most promising virtual hit for pan-AKT inhibition. Regarding the MNK-1 and MNK-2 inhibitors [21], the final MA-mtk model was used for the screening of the commercial library to obtain 20 potential virtual hits. Unlike resorting to reverse pharmacophore mapping to improve confidence over these hits and to select the most promising hits, a much faster strategy based on similarity searching was taken into consideration. In this method, the fingerprints of the virtual hits were cross-matched with a database containing MNK-1 and MNK-2 inhibitors to identify those hits with a maximum number of matches vs. the experimentally tested potent MNK-1/2 inhibitors. These filtered hits were further processed by MD simulation analyses and theoretical binding energy calculations that led to only the four most promising candidates. In future it is expected that experimental validation coupled with these in silico strategies may lead towards finding target-based therapeutic agents for various other diseases.

In addition to the cancer progression targets discussed above, biological targets related to other diseases such as antimicrobial [24,80,81,82], antihypertensive [83], neuroprotective and neurotoxic [13,84], and anti-inflammatory agents [35], and have been the object of research within the MA-mtk-QSAR context. In a recent work aimed at designing anti-inflammatory agents through the dual inhibition of caspase-1 and TNF-α, the dataset contained 1476 data points built from 1444 molecules with activity tested against caspase-1 or TNF-α [35]. Evidently, the data was structurally and biologically diverse in nature and so the authors considered only two experimental conditions, namely the biological targets and their experimental assay types, to derive the deviation descriptors starting from topological indices. Two similar but different cut-off values of 1000 nM and 1635 nM were assigned for these two biological targets to distinguish the active samples from inactive ones. The resulting MLP-ANN non-linear model afforded an overall predictive accuracy higher than 88%, and a virtual screening was performed with agency-regulatory chemicals to select and rank the most promising virtual hits for dual inhibition of these two proteins.

## 5. Software Developed for Multitasking Modeling

This section briefly describes three software packages that have been developed recently for accelerating MA-mtk modeling as outlined in the current review. These are QSAR-Co, QSAR-Co-X and FRAMA. Both QSAR-Co and QSAR-Co-X are available in the public domain with detailed instruction manuals.

### 5.1. QSAR-Co

**Figure 4:**
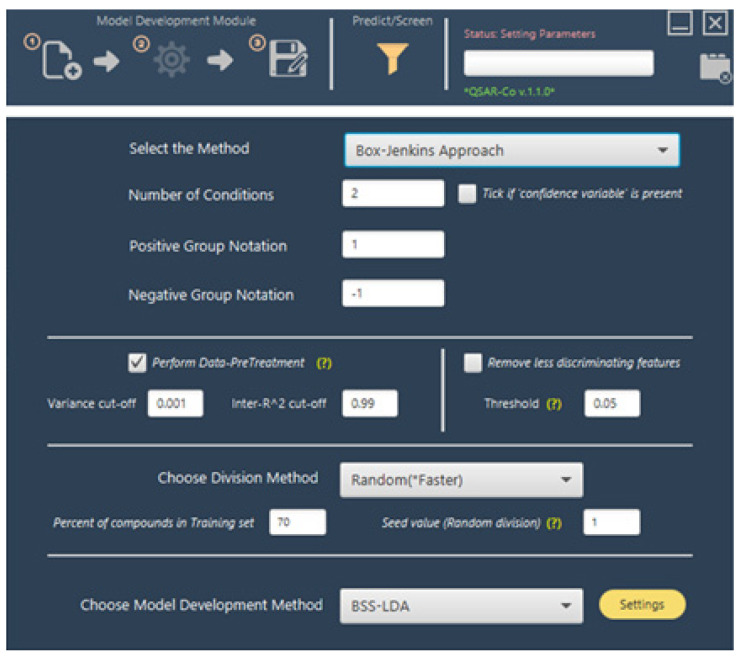
Screenshot of the latest version of QSAR-Co (version 1.1.0) [44].

QSAR-Co [44], which was introduced in 2019, is a Java based open-access tool for developing moving average-based mtk-QSAR models by means of GA-LDA and RF techniques (available at https://sites.google.com/view/qsar-co, see Figure 4). This software, which utilizes the WEKA library for RF-based model development, was designed to automatically calculate moving average-based deviation descriptors starting from the original descriptors, which are fed into the software as a .csv file, alongside the name of compounds, the experimental/theoretical conditions, and the endpoint response(s) to be targeted. The software automatically yields output .csv files containing statistical parameters such as the sensitivity, specificity, accuracy, the Matthews correlation coefficient (MCC), etc., and receiver operating characteristics (ROC) plots of the models, along with the selected features values, observed and predicted response(s), as well as the applicability domain estimated by either the standardization approach or the confidence estimation approach. Furthermore, QSAR-Co allows the remotion of less important descriptors to be performed, the division of the dataset with multiple methods, and is also capable of diagnosing query chemicals, which is extremely useful in virtual screening efforts.

### 5.2. QSAR-Co-X

**Figure 5:**
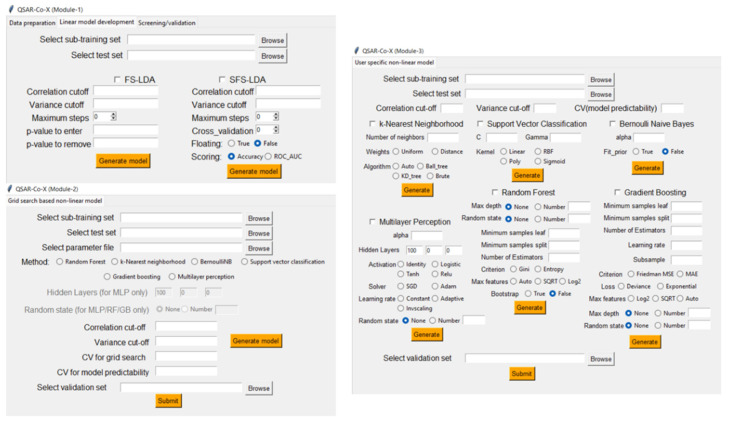
Screenshots of the Modules 1–3 graphic interface from the toolkit QSAR-Co-X [12].

With the aim of expanding the scope of software QSAR-Co, another software named QSAR-Co-X [12] was introduced in 2021. This open source standalone toolkit built by using Python 3 (available at https://github.com/ncordeirfcup/QSAR-Co-X) comprises four different modules. Module 1 is designed for the calculation of deviation descriptors using diverse Box–Jenkins’s operators, starting from the categorical endpoint response(s), the related experimental/theoretical conditions, and the original descriptors. The same module performs data division for generating the training, test and external validation sets, followed by the descriptor generation and development of the linear mtk-QSAR models by application of the LDA technique along with FS or SFS feature-selection algorithms. Subsequently, either prediction when the endpoint response is known or screening when it is unknown can be performed using the external validation set to estimate the ‘true’ predictivity of the model. Furthermore, this module performs *Y*_c_-randomization and produces output files containing the resulting statistical parameters, and the information regarding the model descriptors and its applicability domain determined by either the standardization or the confidence estimation approach (see Figure 5). Modules 2 and 3, on the other hand, are intended for the development of non-linear models using multiple machine learning methods including (a) *k*-nearest neighborhood (*k*NN), (b) Bernoulli naïve Bayes (NB) classifier, (c) support vector classifier (SVC), (d) random forests (RF), (e) gradient boosting (GB), and (f) multilayer perceptron (MLP) neural networks. For all these non-linear modeling techniques, the Scikit-learn machine learning package is used. Module 2 provides the facility of hyperparameter optimization for each of these ML tools based on the information provided by the user in .csv format. On the other hand, non-linear models are developed using the fixed user-specific parameters. Module 4 is used for ‘condition-wise prediction’ to assess the accuracy of the generated models against each experimental condition.

### 5.3. FRAMA

**Figure 6:**
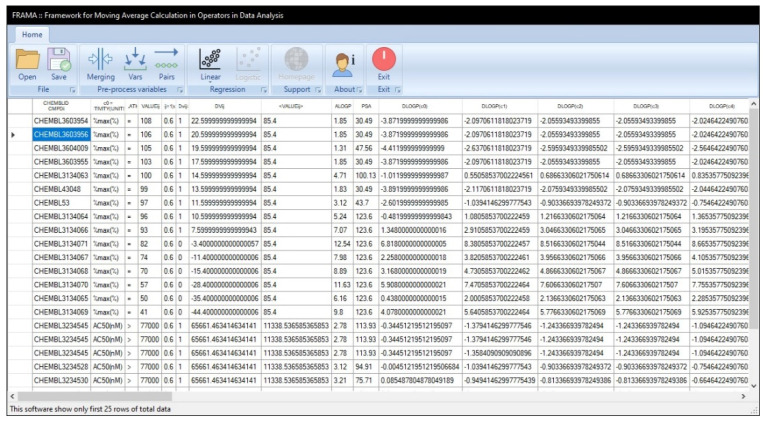
Screenshot of the latest version of the Windows software FRAMA [10,85].

FRAMA, is a Windows desktop application developed in 2017 [10,85], which supports various file formats and allows the user to perform several data preprocessing and classification tasks of the input and output variables (see Figure 6). After pretreating the data, the selected variables can then be subjected to batch operations by the user, in which classical BJMA operators can be computed for conducting multilinear regression or classification multitasking assessments, as well as PTML analyses. The processing information alongside several parametric statistics computed for each type of modeling is stored in .csv spreadsheets for further analysis.

## 6. Future Scope

MA-mtk modeling techniques have been applied, with excellent results, in a number of different research areas. Irrespective of the nature of the chemicals, disease, targets, experimental conditions, or even dataset size, highly predictive models have been obtained. However, as the majority of investigations have been focused on anticancer and antimicrobial research, the likelihood, in coming years, of MA-mtk modeling expanding to cover diseases such as diabetes, cardiovascular disorders, inflammatory disorders, or CNS disorders, where only a few investigations have been performed till date, is to be expected.

In addition, the scope of deep learning has already been discussed in model development, and similarly, model development and validation should be improved with the inclusion of more refined feature selection and ML tools (e.g., logistic regression), which are still witnessing extensive advancement and transformation [86,87]. Further work may also be required on the modification of Box–Jenkin’s moving average algorithms, and comparative analyses should be performed to understand if any of such modification leads to a model with improved predictive accuracy or not.

Another aspect to consider is that investigations have so far focused on fragment-based design [8,27,28], whereas other relied on virtual screening of commercial databases [21,46]. Recently, the application of Bemis–Murcko scaffolds was suggested to extract the fragments from large databases to estimate their contributions [11,46]. However, lead generation and optimization from favorable fragments should be made more systematic in the future by using methods such as scaffold hopping, fragment linking, fragment growing, R-group analyses, or PROTAC design, for example [88]. New techniques, if implemented, made available to the users in the form of tools in software packages such as QSAR-Co, QSAR-Co-X, etc., will allow the advance of the research globally. Finally, experimental validation of the proposed hits must be encouraged to realize the true potential of MA-mtk modeling.

## 7. Conclusions

With increasing chemical and biological knowledge, which produces a huge amount of available data, continuously accumulated in scientific literature and databases, the in silico methods adopted for the design of new molecular entities must be able to tackle in a fast and simple manner this scientific data, if molecular design is to evolve towards a multitasking optimization process. The current review focuses on the current status and future scope of moving average-based multitasking in silico modeling that tend to serve the above-mentioned purpose. Alongside discussing the basic methodologies of MA-mtk modeling, some works were specifically addressed at understanding how their integration of datasets with variable experimental assay conditions improves the diversity and reliability of in silico models. This discussion may provide a more wholistic idea about mechanistic interpretations. Furthermore, the discussion of these recent advances included the newly-developed tools for facilitating such modeling. As such, not only does this review provide important updates and guidelines for multitasking in silico classification modeling but it also explores how it is expected that such modeling will expand the research areas that are yet to be covered.

## Figures and Tables

**Table 1:** Box–Jenkins operators used in the different studies [24,29,30,31,32,33,34,35].

*Operators*	*Remarks*
∆(*D_i_*)*c_j_ = D_i_ − avg*(*D_i_*)*c_j_*	$avg(D_{i})c_{j}=\frac{1}{n(c_{j})}\sum_{i=1}^{n(c_{j})}D_{i}$
∆(*D_i_*)*c_j_ = p_c_*·[*D_i_ − avg*(*D_i_*)*c_j_*]	*p_c_: A probabilistic term*
∆(*D_i_*)*c_j_ =* [*D_i_ − avg*(*D_i_*)*c_j_*]/(*D_imax_ − D_imin_*)	*D_imax_*: *Maximum value of D_i_* *D_imin_:Minimum value of D_i_*
∆(*D_i_*)*c_j_ =* [*D_i_ − avg*(*D_i_*)*c_j_*]/[(*D_imax_ − D_imin_*) *p*(*c_j_*)*_c_*]	*p*(*c_j_*)*_c_ = n*(*c_j_*)/*N* (*N*: Total number of data points in the modeling set)
∆(*D_i_*)*c_j_ =* [*D_i_ − avg*(*D_i_*)*c_j_*]/[(*D_imax_ − D_imin_*) *√p*(*c_j_*)*_c_*]	*p*(*c_j_*)*_c_ = n*(*c_j_*)/N
∆(*D_i_*)*c_j_ =* [(*D_i_ − avg*(*D_i_*)*c_j_*]/[*SD*(*D_i_*) *√p*(*c_j_*)*_c_*]	SD(*D_i_*): Standard deviation of *D_i_*

**Table 2:** Feature selection and machine learning tools used for moving average based multitasking modeling [12,21,44].

Feature Selection Tools—Linear Models (LDA)	Machine Learning Tools—Non-Linear Models
Fast stepwise (FS) selection	Decision trees (DT)
Sequential forward selection (SFS)	Random forests (RF)
Genetic algorithm (GA) selection	Gradient boosting (GB)
Post-selection similarity search modification (PS3M)	Support vector machines (SVM)
	*k*-nearest neighborhood (*k*NN)
	Bernoulli naïve Bayes (NB)
	Artificial neural networks (ANN)
	Deep neural networks (DNN)

**Table 3:** Selected multitasking classification modeling studies in antimicrobial and antiviral research.

Year	Methodology ^a^	No. of Chemicals (*N*_dp_) ^b^	Endpoint Responses ^c^	Bio-Targets ^d^	Acc (%) ^e^	Ref.
2013	RBF-ANN	8560 (10,918)	Anti-Enterococci activities and toxicological profiles	*Enterococci* strains; *Mus musculus*; *Rattus norvegicus*; human lymphocytes	92.30	[59]
2013	RBF-ANN	6974 (11,576)	Anti-Streptococci activities and toxicological profiles	Streptococci strains; *Mus musculus*; *Rattus norvegicus*	98.08	[60]
2013	FS-LDA	20,863 (34,629)	Anti-Mycobacterial activity and ADMET properties	*Mycobacterium* spp. strains; proteins; *Mus musculus*; *Rattus norvegicus*; *Homo sapiens*	94.80	[51]
2014	FS-LDA	23,705 (37,834)	Anti-Escherichia coli activities and ADMET properties	*Escherichia coli* strains; proteins; laboratory animals (mice and rats); *Homo sapiens*	95.85	[52]
2014	FS-LDA	26,945 (48,874)	Anti-cocci activities and ADMET properties	Gram-positive cocci strains; proteins; cell lines; laboratory animals; humans	92.89	[61]
2014	LNN-LDA	21,582 (43,249)	Anti-HIV-1 activity and epidemiological profile	Viral or human proteins/enzymes (e.g., CC-CKR-5, HIV-1 RT, and HIV-1 PR); laboratory animals; humans	76.76	[62]
2015	FS-LDA	30,738 (54,682)	Anti-*Pseudomonas* activities and ADMET properties	*Pseudomonas* spp. strains; proteins/enzymes; *Mus musculus*; *Rattus norvegicus*; *Homo sapiens*	90.62	[63]
2015	FS-LDA	22,009 (30,181)	Anti-NOMA activity and ADMET profiles	Bacteria linked to NOMA infections (e.g., *Fusobacterium* spp., *Prevotella* spp., *Bacillus*, etc.); cell lines; laboratory animals; humans	92.12	[53]
2016	FS-LDA	2123 (3592)	Anti-microbial peptides (AMP) activity and cytotoxicity	Gram-negative bacterial strains; mammalian cell types	97.40	[50]
2016	FS-LDA	1581 (2488)	AMP activity	Gram-positive bacterial strains	94.57	[64]
2017	FS-LDA	20,562 (29,682)	Anti-HIV activity and ADMET properties	HIV; proteins/enzymes; cell lines; laboratory animals; humans	96.26	[43]
2017	FS-LDA	29,863 (40,158)	Anti-Hepatitis C activity and ADMET properties	Hepatitis C; proteins/enzymes; mammalian cells	95.35	[31]
2020	MLP-ANN	18,798 (21,369)	Anti-malarial activity, cytotoxicity, and pharmacokinetic properties	*Plasmodium falciparum* strains; proteins; mammalian cells; plasma and liver microsomes	90.49	[65]

**^a^** RBF: radial basis function; ANN: artificial neural networks; FS-LDA: forward stepwise–linear discriminant analysis; LNN: linear neural networks; MLP: multilayer perceptron. ^b^ No. of chemicals: Number of chemicals with unique structures; *N*_dp_: Number of data points considered in the modeling taking into account the experimental conditions. **^c^** ADMET: absorption, distribution, metabolism, elimination, and toxicity; AMP: antimicrobial peptides. ^d^ CC-CKR-5: C−C chemokine receptor type 5; HIV-1 RT: HIV-1 reverse transcriptase; HIV-1 PR: HIV-1 protease; NOMA: cancrum oris; Mtb: *Mycobacterium tuberculosis*. ^e^ Average accuracy obtained from the training and prediction sets.

**Table 4:** Some in silico tools and webservers employed for multitasking modeling.

Method	Software/Webserver
Pharmacophore mapping	PharmMapper [25,71]
Molecular docking	AutoDock [21,25,72], AutoDock Vina [25,73], Molegro Virtual Docker [24,74]
Similarity search	SIMSEARCH [21]
Molecular dynamics simulations	Amber [21,75], Gromacs [46,76]
Homology modeling	SwissModel [25,77]
Drug-likeness	SwissADME [21,78]
Synthetic accessibility	SwissADME [21,78]
Graph-based signature	MycoCSM [22,79]

## Data Availability

Not applicable.

## References

[1] Hansch C., Maloney P.P., Fujita T., Muir R.M. (1962). Correlation of Biological Activity of Phenoxyacetic Acids with Hammett Substituent Constants and Partition Coefficients. Nature.

[2] Muratov E.N., Bajorath J., Sheridan R.P., Tetko I.V., Filimonov D., Poroikov V., Oprea T.I., Baskin I.I., Varnek A., Roitberg A. (2020). QSAR Without Borders. Chem. Soc. Rev..

[3] Neves B.J., Braga R.C., Melo-Filho C.C., Moreira-Filho J.T., Muratov E.N., Andrade C.H. (2018). QSAR-Based Virtual Screening: Advances and Applications in Drug Discovery. Front. Pharmacol..

[4] Yu W., MacKerell A.D., Sass P. (2017). Computer-Aided Drug Design Methods. Antibiotics. Methods in Molecular Biology.

[5] Abdolmaleki A., Ghasemi J., Ghasemi F. (2017). Computer Aided Drug Design for Multi-Target Drug Design: SAR/QSAR, Molecular Docking and Pharmacophore Methods. Curr. Drug Targets.

[6] Sabe V.T., Ntombela T., Jhamba L.A., Maguire G.E.M., Govender T., Naicker T., Kruger H.G. (2021). Current trends in computer aided drug design and a highlight of drugs discovered via computational techniques: A review. Eur. J. Med. Chem..

[7] Halder A.K., Moura A.S., Cordeiro M.N.D.S. (2018). QSAR modelling: A therapeutic patent review 2010-present. Expert Opin. Ther. Pat..

[8] Speck-Planche A. (2018). Recent advances in fragment-based computational drug design: Tackling simultaneous targets/biological effects. Future Med. Chem..

[9] Searls D.B. (2005). Data integration: Challenges for drug discovery. Nat. Rev. Drug Discov..

[10] Ortega-Tenezaca B., Quevedo-Tumailli V., Bediaga H., Collados J., Arrasate S., Madariaga G., Munteanu C.R., Cordeiro M.N.D.S., Gonzalez-Díaz H. (2020). PTML Multi-Label Algorithms: Models, Software, and Applications. Curr. Top. Med. Chem..

[11] Halder A.K., Cordeiro M.N.D.S. (2019). Development of Multi-Target Chemometric Models for the Inhibition of Class I PI3K Enzyme Isoforms: A Case Study Using QSAR-Co Tool. Int. J. Mol. Sci..

[12] Halder A.K., Cordeiro M.N.D.S. (2021). QSAR-Co-X: An open source toolkit for multitarget QSAR modelling. J. Cheminform..

[13] Speck-Planche A., Cordeiro M.N.D.S. (2015). Multitasking models for quantitative structure–biological effect relationships: Current status and future perspectives to speed up drug discovery. Expert Opin. Drug Discov..

[14] Lambrinidis G., Tsantili-Kakoulidou A. (2020). Multi-objective optimization methods in novel drug design. Expert Opin. Drug Discov..

[15] Speck-Planche A. (2019). Multi-Scale Modeling in Drug Discovery against Infectious Diseases. Mini Rev. Med. Chem..

[16] Toropov A.A., Toropova A.P. (2020). QSPR/QSAR: State-of-Art, Weirdness, the Future. Molecules.

[17] Roy K., Kar S., Das R.N. (2015). Chemical Information and Descriptors. Understanding the Basics of QSAR for Applications in Pharmaceutical Sciences and Risk Assessment.

[18] Halder A.K., Delgado A.H.S., Cordeiro M.N.D.S. (2022). First multi-target QSAR model for predicting the cytotoxicity of acrylic acid-based dental monomers. Dental Mater..

[19] Prado-Prado F.J., Gonzalez-Díaz H., de la Vega O.M., Ubeira F.M., Chou K.-C. (2008). Unified QSAR approach to antimicrobials. Part 3: First multi-tasking QSAR model for input-coded prediction, structural back-projection, and complex networks clustering of antiprotozoal compounds. Bioorg. Med. Chem..

[20] Kleandrova V.V., Speck-Planche A. (2021). The urgent need for pan-antiviral agents: From multitarget discovery to multiscale design. Future Med. Chem..

[21] Halder A.K., Cordeiro M.N.D.S. (2021). Multi-Target in Silico Prediction of Inhibitors for Mitogen-Activated Protein Kinase-Interacting Kinases. Biomolecules.

[22] Kleandrova V.V., Scotti M.T., Speck-Planche A. (2021). Computational Drug Repurposing for Antituberculosis Therapy: Discovery of Multi-Strain Inhibitors. Antibiotics.

[23] Cumming J.G., Davis A.M., Muresan S., Haeberlein M., Chen H. (2013). Chemical predictive modelling to improve compound quality. Nat. Rev. Drug Discov..

[24] Kleandrova V.V., Scotti L., Junior F.J.B.M., Muratov E., Scotti M.T., Speck-Planche A. (2021). QSAR Modeling for Multi-Target Drug Discovery: Designing Simultaneous Inhibitors of Proteins in Diverse Pathogenic Parasites. Front. Chem..

[25] Halder A.K., Cordeiro M.N.D.S. (2021). AKT Inhibitors: The Road Ahead to Computational Modeling-Guided Discovery. Int. J. Mol. Sci..

[26] Box G.E.P., Jenkins G.M., Reinsel G.C., Ljung G.M. (1970). Time Series Analysis: Forecasting and Control.

[27] Speck-Planche A., Kleandrova V.V., Luan F., Cordeiro M.N.D.S. (2011). Fragment-based QSAR model toward the selection of versatile anti-sarcoma leads. Eur. J. Med. Chem..

[28] Speck-Planche A., Kleandrova V.V., Scotti M.T. (2012). Fragment-based approach for the in silico discovery of multi-target insecticides. Chemom. Intell. Lab. Syst..

[29] Halder A.K., Haghbakhsh R., Voroshylova I.V., Duarte A.R.C., Cordeiro M.N.D.S. (2021). Density of Deep Eutectic Solvents: The Path Forward Cheminformatics-Driven Reliable Predictions for Mixtures. Molecules.

[30] Speck-Planche A., Cordeiro M.N.D.S. (2017). De novo computational design of compounds virtually displaying potent antibacterial activity and desirable in vitro ADMET profiles. Med. Chem. Res..

[31] Speck-Planche A., Cordeiro M.N.D.S. (2017). Speeding up Early Drug Discovery in Antiviral Research: A Fragment-Based in Silico Approach for the Design of Virtual Anti-Hepatitis C Leads. ACS Comb. Sci..

[32] Kleandrova V.V., Scotti M.T., Scotti L., Nayarisseri A., Speck-Planche A. (2020). Cell-based multi-target QSAR model for design of virtual versatile inhibitors of liver cancer cell lines. SAR QSAR Environ. Res..

[33] Speck-Planche A., Roy K. (2020). Multi-Scale QSAR Approach for Simultaneous Modeling of Ecotoxic Effects of Pesticides. Ecotoxicological QSARs. Methods in Pharmacology and Toxicology.

[34] Kleandrova V.V., Scotti M.T., Scotti L., Speck-Planche A. (2021). Multi-target Drug Discovery via PTML Modeling: Applications to the Design of Virtual Dual Inhibitors of CDK4 and HER2. Curr. Top. Med. Chem..

[35] Speck-Planche A., Kleandrova V.V., Scotti M.T. (2021). In Silico Drug Repurposing for Anti-Inflammatory Therapy: Virtual Search for Dual Inhibitors of Caspase-1 and TNF-Alpha. Biomolecules.

[36] Valdés-Martiní J.R., Marrero-Ponce Y., García-Jacas C.R., Martinez-Mayorga K., Barigye S.J., Vaz d‘Almeida Y.S., Pham-The H., Pérez-Giménez F., Morell C.A. (2017). QuBiLS-MAS, open source multi-platform software for atom- and bond-based topological (2D) and chiral (2.5D) algebraic molecular descriptors computations. J. Cheminform..

[37] García I., Fall Y., Gómez G., Gonzalez-Díaz H. (2010). First computational chemistry multi-target model for anti-Alzheimer, anti-parasitic, anti-fungi, and anti-bacterial activity of GSK-3 inhibitors in vitro, in vivo, and in different cellular lines. Mol. Divers..

[38] Speck-Planche A., Scotti M.T. (2018). BET bromodomain inhibitors: Fragment-based in silico design using multi-target QSAR models. Mol. Divers..

[39] Mauri A., Consonni V., Pavan M., Todeschini R. (2006). DRAGON software: An easy approach to molecular descriptor calculations. MATCH Commun. Math. Comput. Chem..

[40] Kleandrova V.V., Speck-Planche A. (2022). PTML Modeling for Pancreatic Cancer Research: In Silico Design of Simultaneous Multi-Protein and Multi-Cell Inhibitors. Biomedicines.

[41] Speck-Planche A. (2018). Combining Ensemble Learning with a Fragment-Based Topological Approach to Generate New Molecular Diversity in Drug Discovery: In Silico Design of Hsp90 Inhibitors. ACS Omega.

[42] Speck-Planche A. (2019). Multicellular Target QSAR Model for Simultaneous Prediction and Design of Anti-Pancreatic Cancer Agents. ACS Omega.

[43] Kleandrova V.V., Speck-Planche A., Speck-Planche A. (2017). Multitasking Model for Computer-Aided Design and Virtual Screening of Compounds with High Anti-HIV Activity and Desirable ADMET Properties. Multi-Scale Approaches in Drug Discovery.

[44] Ambure P., Halder A.K., Gonzalez Diaz H., Cordeiro M.N.D.S. (2019). QSAR-Co: An Open Source Software for Developing Robust Multitasking or Multitarget Classification-Based QSAR Models. J. Chem. Inf. Model..

[45] Urias R.W.P., Barigye S.J., Marrero-Ponce Y., García-Jacas C.R., Valdes-Martiní J.R., Perez-Gimenez F. (2015). IMMAN: Free software for information theory-based chemometric analysis. Mol. Divers..

[46] Halder A.K., Giri A.K., Cordeiro M.N.D.S. (2019). Multi-Target Chemometric Modelling, Fragment Analysis and Virtual Screening with ERK Inhibitors as Potential Anticancer Agents. Molecules.

[47] Roy K., Kar S., Das R.N. (2015). Validation of QSAR Models. Understanding the Basics of QSAR for Applications in Pharmaceutical Sciences and Risk Assessment.

[48] Roy K., Kar S., Ambure P. (2015). On a simple approach for determining applicability domain of QSAR models. Chemom. Intell. Lab. Syst..

[49] Ambure P., Bhat J., Puzyn T., Roy K. (2018). Identifying natural compounds as multi-target-directed ligands against Alzheimer’s disease: An in silico approach. J. Biomol. Struct. Dyn..

[50] Kleandrova V.V., Ruso J.M., Speck-Planche A., Cordeiro M.N.D.S. (2016). Enabling the Discovery and Virtual Screening of Potent and Safe Antimicrobial Peptides. Simultaneous Prediction of Antibacterial Activity and Cytotoxicity. ACS Comb. Sci..

[51] Speck-Planche A., Cordeiro M.N.D.S. (2013). Simultaneous Modeling of Antimycobacterial Activities and ADMET Profiles: A Chemoinformatic Approach to Medicinal Chemistry. Curr. Top. Med. Chem..

[52] Speck-Planche A., Cordeiro M.N.D.S. (2014). Simultaneous Virtual Prediction of Anti-Escherichia coli Activities and ADMET Profiles: A Chemoinformatic Complementary Approach for High-Throughput Screening. ACS Comb. Sci..

[53] Speck-Planche A., Cordeiro M.N.D.S. (2015). Enabling Virtual Screening of Potent and Safer Antimicrobial Agents against Noma: Mtk-QSBER Model for Simultaneous Prediction of Antibacterial Activities and ADMET Properties. Mini Rev. Med. Chem..

[54] Speck-Planche A., Kleandrova V.V., Luan F., Cordeiro M.N.D.S. (2011). Multi-target drug discovery in anti-cancer therapy: Fragment-based approach toward the design of potent and versatile anti-prostate cancer agents. Bioorg. Med. Chem..

[55] Speck-Planche A., Kleandrova V.V., Luan F., Cordeiro M.N.D.S. (2012). Chemoinformatics in anti-cancer chemotherapy: Multi-target QSAR model for the in silico discovery of anti-breast cancer agents. Eur. J. Pharm. Sci..

[56] Speck-Planche A., Kleandrova V.V., Luan F., Cordeiro M.N.D.S. (2012). Chemoinformatics in Multi-target Drug Discovery for Anti-cancer Therapy: In Silico Design of Potent and Versatile Anti-brain Tumor Agents. Anti-Cancer Agents Med. Chem..

[57] Speck-Planche A., Kleandrova V.V., Luan F., Cordeiro M.N.D.S. (2012). Rational drug design for anti-cancer chemotherapy: Multi-target QSAR models for the in silico discovery of anti-colorectal cancer agents. Bioorg. Med. Chem..

[58] Speck-Planche A., Kleandrova V.V., Luan F., Cordeiro M.N.D.S. (2013). Unified Multi-target Approach for the Rational in silico Design of Anti-bladder Cancer Agents. Anti Cancer Agents Med. Chem..

[59] Speck-Planche A., Cordeiro M.N.D.S. Chemoinformatics in Drug Design. Artificial Neural Networks for Simultaneous Prediction of Anti-Enterococci Activities and Toxicological Profiles. Proceedings of the 5th International Joint Conference on Computational Intelligence.

[60] Speck-Planche A., Kleandrova V.V., Cordeiro M.N.D.S. (2013). Chemoinformatics for rational discovery of safe antibacterial drugs: Simultaneous predictions of biological activity against streptococci and toxicological profiles in laboratory animals. Bioorg. Med. Chem..

[61] Speck-Planche A., Cordeiro M.N.D.S. (2014). Chemoinformatics for medicinal chemistry: In silico model to enable the discovery of potent and safer anti-cocci agents. Future Med. Chem..

[62] Gonzalez-Díaz H., Herrera-Ibatá D.M., Duardo-Sánchez A., Munteanu C.R., Orbegozo-Medina R.A., Pazos A. (2014). ANN Multiscale Model of Anti-HIV Drugs Activity vs. AIDS Prevalence in the US at County Level Based on Information Indices of Molecular Graphs and Social Networks. J. Chem. Inf. Mod..

[63] Speck-Planche A., Cordeiro M.N.D.S. (2015). Computer-Aided Discovery in Antimicrobial Research: In Silico Model for Virtual Screening of Potent and Safe Anti-Pseudomonas Agents. Comb. Chem. High Throughput Screen.

[64] Speck-Planche A., Kleandrova V.V., Ruso J.M., Cordeiro M.N.D.S. (2016). First Multitarget Chemo-Bioinformatic Model to Enable the Discovery of Antibacterial Peptides against Multiple Gram-Positive Pathogens. J. Chem. Inf. Model..

[65] Speck-Planche A., Kleandrova V.V., Cartwright H.M. (2020). Demystifying Artificial Neural Networks as Generators of New Chemical Knowledge: Antimalarial Drug Discovery as a Case Study. Machine Learning in Chemistry.

[66] Kleandrova V.V., Luan F., Gonzalez-Díaz H., Ruso J.M., Melo A., Speck-Planche A., Cordeiro M.N.D.S. (2014). Computational ecotoxicology: Simultaneous prediction of ecotoxic effects of nanoparticles under different experimental conditions. Environ. Int..

[67] Halder A.K., Cordeiro M.N.D.S. (2019). Probing the Environmental Toxicity of Deep Eutectic Solvents and Their Components: An In Silico Modeling Approach. ACS Sustain. Chem. Eng..

[68] Marzaro G., Chilin A., Guiotto A., Uriarte E., Brun P., Castagliuolo I., Tonus F., Gonzalez-Díaz H. (2011). Using the TOPS-MODE approach to fit multi-target QSAR models for tyrosine kinases inhibitors. Eur. J. Med. Chem..

[69] Speck-Planche A., Luan F., Cordeiro M.N.D.S. (2013). Abelson Tyrosine-Protein Kinase 1 as Principal Target for Drug Discovery Against Leukemias. Role of the Current Computer-Aided Drug Design Methodologies. Curr. Top. Med. Chem..

[70] Casañola-Martin G.M., Le-Thi-Thu H., Pérez-Giménez F., Marrero-Ponce Y., Merino-Sanjuán M., Abad C., Gonzalez-Díaz H. (2015). Multi-output model with Box–Jenkins’s operators of linear indices to predict multi-target inhibitors of ubiquitin–proteasome pathway. Mol. Divers..

[71] Liu X.F., Ouyang S.S., Yu B.A., Liu Y.B., Huang K., Gong J.Y., Zheng S.Y., Li Z.H., Li H.L., Jiang H.L. (2010). PharmMapper server: A web server for potential drug target identification using pharmacophore mapping approach. Nucleic Acids Res..

[72] Morris G.M., Huey R., Lindstrom W., Sanner M.F., Belew R.K., Goodsell D.S., Olson A.J. (2009). AutoDock4 and AutoDockTools4: Automated docking with selective receptor flexibility. J. Comput. Chem..

[73] Trott O., Olson A.J. (2010). Software news and update AutoDock Vina: Improving the speed and accuracy of docking with a new scoring function, efficient optimization, and multithreading. J. Comput. Chem..

[74] Thomsen R., Christensen M.H. (2006). MolDock: A new technique for high-accuracy molecular docking. J. Med. Chem..

[75] Case D.A., Cheatham T.E., Darden T., Gohlke H., Luo R., Merz K.M., Onufriev A., Simmerling C., Wang B., Woods R.J. (2005). The Amber biomolecular simulation programs. J. Comput. Chem..

[76] Hess B., Kutzner C., van der Spoel D., Lindahl E. (2008). GROMACS 4: Algorithms for highly efficient, load-balanced, and scalable molecular simulation. J. Chem. Theory Comput..

[77] Guex N., Peitsch M.C. (1997). SWISS-MODEL and the Swiss-PdbViewer: An environment for comparative protein modeling. Electrophoresis.

[78] Daina A., Michielin O., Zoete V. (2017). SwissADME: A free web tool to evaluate pharmacokinetics, drug-likeness and medicinal chemistry friendliness of small molecules. Sci. Rep..

[79] Pires D.E.V., Ascher D.B. (2020). mycoCSM: Using Graph-Based Signatures to Identify Safe Potent Hits against Mycobacteria. J. Chem. Inf. Model..

[80] Speck-Planche A., Cordeiro M.N.D.S. (2015). Multi-Target QSAR Approaches for Modeling Protein Inhibitors. Simultaneous Prediction of Activities against Biomacromolecules Present in Gram-Negative Bacteria. Curr. Top. Med. Chem..

[81] Kleandrova V.V., Luan F., Speck-Planche A., Cordeiro M.N.D.S. (2015). Review of Structures Containing Fullerene-C60 for Delivery of Antibacterial Agents. Multitasking model for Computational Assessment of Safety Profiles. Curr. Bioinform..

[82] Speck-Planche A., Kleandrova V.V. (2011). In silico design of multi-target inhibitors for C–C chemokine receptors using substructural descriptors. Mol. Divers..

[83] Kleandrova V.V., Rojas-Vargas J.A., Scotti M.T., Speck-Planche A. (2021). PTML modeling for peptide discovery: In silico design of non-hemolytic peptides with antihypertensive activity. Mol. Divers..

[84] Speck-Planche A., Luan F., Cordeiro M.N.D.S. (2012). Role of Ligand-Based Drug Design Methodologies toward the Discovery of New Anti-Alzheimer Agents: Futures Perspectives in Fragment-Based Ligand Design. Curr. Med. Chem..

[85] Gonzalez-Diaz H., Ortega-Tenezaca B., Quevedo-Tumailli V. FRAMA 1.0: Framework for Moving Average Operators Calculation in Data Analysis. Proceedings of the MOL2NET 2017, International Conference on Multidisciplinary Sciences, 3rd Ed.

[86] Le N.Q.K., Kha Q.H., Nguyen V.H., Chen Y.-C., Cheng S.-J., Chen C.-Y. (2021). Machine Learning-Based Radiomics Signatures for EGFR and KRAS Mutations Prediction in Non-Small-Cell Lung Cancer. Int. J. Mol. Sci..

[87] Hung T.N.K., Le N.Q.K., Le N.H., Tuan L.V., Nguyen T.P., Thi C., Kang J.H. (2022). An AI-based Prediction Model for Drug-drug Interactions in Osteoporosis and Paget’s Diseases from SMILES. Mol. Inform..

[88] Imrie F., Hadfield T.E., Bradley A.R., Deane C.M. (2021). Deep generative design with 3D pharmacophoric constraints. Chem. Sci..

